# Care Aides’ Perceptions of Caring for Nursing Home Residents With Past Psychological Trauma

**DOI:** 10.1093/geroni/igab046.3097

**Published:** 2021-12-17

**Authors:** Trina Thorne, Heather Titley, Peter Norton, Ruth Lanius, Carole Estabrooks

**Affiliations:** 1 University of Alberta, Edmonton, Alberta, Canada; 2 University of Calgary, University of Calgary, Alberta, Canada; 3 University of Western Ontario, London, Ontario, Canada

## Abstract

The dynamic interplay between dementia and psychological trauma can exert powerful effects on nursing home residents’ behavioral symptoms and quality of life. Our objectives in this exploratory study were to assess care aides’ perceptions of how often they worked with residents with past psychological trauma, the types of trauma encountered, and reasons for these beliefs. We conducted semi-structured cognitive interviews (n = 10) with care aides in June 2019 to inform the development of a trauma needs assessment (4 questions) that we included in a large survey of nursing staff (2019 - 2020). Care aides (n = 3761) were sampled from 91 randomly selected urban nursing homes stratified by health region, owner operator model, and size. We completed basic statistics and content analyses. Care aides identified residents they believed to have psychological trauma histories and provided reasons for their beliefs. Approximately 12% of the reported traumatic events were disclosed to staff. The most common, broad categories of trauma to emerge during analysis were abuse (40%) and war exposure (30%). Each had sub-categories. The most common categories of signs of trauma were re-experiencing symptoms such as flashbacks and nightmares (28%), and avoidance of specific triggers, such as water or intimate care (24%). The majority of the reported signs of trauma were persistent and distressing for staff and residents. Some behaviours assumed to be responsive behaviours of dementia may relate to traumatic stress symptomatology. Implementing trauma-informed supports for residents and care aides is essential to person-centred care and optimal quality of life.

